# Altered microbiota associated with abnormal humoral immune responses to commensal organisms in enthesitis-related arthritis

**DOI:** 10.1186/s13075-014-0486-0

**Published:** 2014-11-30

**Authors:** Matthew L Stoll, Ranjit Kumar, Casey D Morrow, Elliot J Lefkowitz, Xiangqin Cui, Anna Genin, Randy Q Cron, Charles O Elson

**Affiliations:** Department of Pediatric Rheumatology, University of Alabama at Birmingham, CPP N 210M, 1600 7th Avenue South, Birmingham, AL 35233 USA; Center for Clinical and Translational Sciences, University of Alabama at Birmingham, CPP N 210M, 1720 2nd Avenue South, Birmingham, AL 35294 USA; Cell, Developmental, and Integrative Biology, University of Alabama at Birmingham, CPP N 210M, 1720 2nd Avenue South, Birmingham, AL 35294 USA; Department of Microbiology, University of Alabama at Birmingham, CPP N 210M, 1720 2nd Avenue South, Birmingham, AL 35294 USA; Department of Biostatistics, University of Alabama at Birmingham, CPP N 210M, 1720 2nd Avenue South, Birmingham, AL 35294 USA; Department of Medicine – Gastroenterology, University of Alabama at Birmingham, CPP N 210M, 1720 2nd Avenue South, Birmingham, AL 35294 USA

## Abstract

**Introduction:**

Prior studies have established altered microbiota and immunologic reactivity to enteric commensal organisms in inflammatory bowel disease (IBD). Since intestinal inflammation is present in a subset of patients with both pediatric and adult spondyloarthritis (SpA), we hypothesized that SpA patients may also have altered microbiota and immune responsiveness to enteric organisms.

**Methods:**

Stool and blood specimens were collected from children with enthesitis-related arthritis (ERA) and non-inflammatory controls. DNA purified from stool was subject to PCR amplification and sequencing of the variable IV region from the 16S rDNA gene. IgA and IgG Enzyme-linked Immunosorbent Assays (ELISAs) were performed on select species of bacteria in most subjects.

**Results:**

Twenty-five children with ERA and 13 controls were included. The ERA patients had less *Faecalibacterium prausnitzii* (3.8% versus 10%, *P* = 0.008) and *lachnospiraceae* family (12 versus 7.0%, *P* = 0.020), a statistically significant increase in *bifidobacterium* (1.8% versus 0%, *P* = 0.032) and a non-statistically significant increase in *Bacteroides* (21% versus 11%, *P* = 0.150). *Akkermansia muciniphila* was abundant (>2%) in 7/27 ERA patients but none of the controls (*P* = 0.072.) Cluster analysis revealed two clusters of ERA patients: Cluster one (*n* = 8) was characterized by high levels of *Bacteroides* genus, while a second (*n* = 15) cluster had similar levels as the controls. Seven of 17 (41%) of the ERA subjects in Cluster 2 compared to 0/8 of the subjects in Cluster 1 had abundant *Akkermansia muciniphila* (*P* = 0.057). Serum IgA and IgG antibody levels against *F. prausnitzii* and *B. fragilis* were similar between patients and controls, whereas the two groups showed divergent responses when the fecal relative abundances of *F. prausnitzii* and *Bacteroides* were compared individually against IgA antibody levels recognizing *F. prausnitzii* and *B. fragilis,* respectively.

**Conclusion:**

The abundance of *F. prausnitzii* in the stool among patients with ERA is reduced compared to controls, and *Bacteroides* and *A. muciniphila* are identified as associative agents in subsets of ERA patients. Differences in the humoral responses to these bacteria may contribute to disease.

**Electronic supplementary material:**

The online version of this article (doi:10.1186/s13075-014-0486-0) contains supplementary material, which is available to authorized users.

## Introduction

Spondyloarthritis (SpA) affects up to 1% of the population in the United States [1]. Recent genetic advances have shed some light on pathogenesis and have shown important overlap with inflammatory bowel disease (IBD) [2,3]; however, there still remain important environmental components. Given the association between gut inflammation and SpA [4], one potential environmental trigger that has long attracted attention is the microbiota; indeed, the disease is abrogated in an HLA-B27 transgenic rodent model of colitis and spondylitis when the animals are raised in a germ-free environment [5].

Advances in technology, collectively known as next-generation sequencing, have dramatically lowered the costs of sequencing and thus permitted collections of vast amounts of data, as many as one billion short sequences, in one run [6]. One of the applications has been the assessment of the entire enteric microbiome from multiple individuals. Studies have shown altered microbiota in a variety of inflammatory conditions, including IBD, rheumatoid arthritis (RA), type I diabetes, and celiac disease [7]. Using a variety of molecular approaches, Stebbings *et al*. [8] demonstrated possible decreases in the *Bacteroides-Provotella* and *Clostridium leptum* groups, with an increase in *Bifidobacterium* among ankylosing spondylitis (AS) patients compared to controls, although the overall differences were not statistically significant [8]. As this study did not use next generation sequencing technology, the nature of the microbiota in SpA patients has yet to be fully assessed.

Intestinal bacteria need not be present in abnormal quantities to trigger arthritis; it is also possible that a pathologic immune response to normal resident microbes may result in disease. Fifty percent of patients with Crohn’s disease (CD) express antibodies directed against flagellins of the microbiota, with these antibodies associated with stricturing disease [9]. Flagellin antibodies have also been identified in SpA patients, albeit at lower titers [10]. It is unknown whether additional bacteria may be targeted in SpA patients; even in CD, there is evidence of seroreactivity to other antigens of the microbiota [11]. Additionally, Stebbings *et al*. [8] demonstrated decreased IgA antisera to *Bacteroides vulgatus* among AS patients, despite increased T cell proliferative responses to autologous *Bacteroides* [8]; the same group subsequently demonstrated decreased production of the regulatory cytokine IL-10 by peripheral blood mononuclear cells of AS patients compared to controls [12]. Thus, altered immunologic reactivity to commensal organisms, possibly including *Bacteroides*, may also play an important role in SpA. Herein, sequencing of the 16S ribosomal DNA (rDNA) of patients with enthesitis-related arthritis (ERA; juvenile SpA) and controls was performed, followed by assessment of antigenic reactivity against bacteria differentially present in the patients versus the controls. We hypothesized that ERA patients may have altered microbiota affecting immune responsiveness to enteric microbiota.

## Methods

### Patients

This study was conducted at a single hospital, Children’s of Alabama (CoA). SpA was defined according to the International League of Associations for Rheumatology criteria for juvenile idiopathic arthritis subtype, ERA [13]. Controls came from the following sources: 1) children referred to rheumatology clinic, but found to have non-inflammatory causes of joint pain or irrelevant laboratory values (for example, positive antinuclear antibody (ANA) in a well child [14]), and 2) children from the community identified through advertisements or parental affiliation with CoA. Subjects with recent (within 6 months) use of systemic antibiotic therapy were excluded. Clinical, demographic, and laboratory data were obtained through chart review and use of case report forms. Informed consent was obtained from guardians as well as subjects age 14 years and older; assent was obtained from children ages 7 to 13 years. Institutional Review Board approval was obtained from the University of Alabama at Birmingham.

### Preparation and sequencing of 16S rDNA from fecal material

Subjects collected the samples at home and immediately placed the samples in a 50-ml container filled with Carey-Blair media [15]. Samples were shipped overnight via commercial carrier to our laboratory. Microbial genomic DNA was isolated by standard methods using a kit from Zymo Research (Irvine, CA, USA) as per the manufacturer’s instructions. The purified DNA underwent PCR amplification using primers designed to the conserved region flanking the variable IV region from the 16S rDNA gene, as per Caporaso *et al*. [16]. Purification of the resulting PCR product was performed with a Qiagen kit (Valencia, CA, USA) as per the manufacturer’s instructions. A second purification with Agencout AmPure beads (Danvers, MA, USA) was performed to increase the quality of the DNA amplicons. The PCR products were multiplexed and diluted for cluster generation using the cBot on a single read v.3 flowcell for the HiSeq2000 (Illumina, San Diego, CA, USA); a few samples were run on the MiSeq (Illumina, San Diego, CA, USA), with the sequences truncated to 101 base pairs to match that of the HiSeq output. Fastq conversion of the raw data files was performed following demultiplexing. Quality control of the fastq files was performed with the FASTX toolkit [17]. The remainder of the steps was performed with the Quantitative Insight into Microbial Ecology (QIIME) suite, version 1.7 [16]. Operational taxonomic units (OTUs) were picked with uclust [18] at 99% similarity. Taxonomic assignments to OTUs were performed with the Ribosomal Database Project classifier [19], which was trained with the May 2013 version of the Greengenes 16S database [20]. Multiple sequence alignment of OTUs was performed with PyNAST [21]. Within-sample (alpha) diversity measures were calculated with Shannon and Phylogenetic Diversity [22], and beta-diversity was calculated with Unifrac [23]. Principal coordinates analysis (PCoA) was performed by QIIME to visualize the dissimilarity matrix (beta-diversity) among all the samples, such that samples that are more similar with respect to their taxanomic distribution are closer together in space than samples that are less similar. To assess the statistical significance of the clusters observed from the PCoA, the R package pvclust was used [24]. Pvclust performed hierarchical clustering using the correlation distance matrix; to estimate stability of the resulting clusters, it performed bootstrap analysis at 10,000 iterations, each time using different subsets of the original data (with replacement.) The output yields the approximate unbiased (AU) and the bootstrap probability (BP) values of each cluster, with the former said to be less subject to bias as per the original description [24].

### ELISA

Serum samples obtained at approximately the time of the collection of the stool specimens were centrifuged and stored at −80°C until ready for use. ELISA was performed to measure IgA or IgG reactivity in 96-well flat-bottom Costar plates (Fisher, Rockville, MD, USA). In preliminary studies, amounts of lyophilized bacteria antigen and serum dilutions were optimized to identify parameters that permitted reproducible results that differentiated serum samples from negative controls. The optimized amounts of bacterial lysate antigen were 50 ng for *Bacteroides fragilis* strain 086-5443-2-2 (kindly donated by Cindy Sears), 250 ng for *Akkermansia muciniphila* (ATCC # BAA-835), and 100 ng for *Faecalibacterium prausnitzii* (ATCC # 27766). Plates were coated with the specified amount of antigen in 50 μl of PBS for four hours at 37°C, then were transferred to store at 4°C overnight. After blocking with 5% milk, serum was added in duplicate at a 1:50 dilution for two hours at room temperature, followed by the addition of anti-human IgG or IgA conjugated to peroxidase (Sigma, St Louis, MO, USA) at a 1:10,000 dilution overnight at 4°C. Plates were developed with para-nitrophenyl phosphate and scanned at the 490 nm wavelength.

### Statistical analysis

Comparisons of individual taxa were performed with the Mann-Whitney *U*-test for two groups and the Kruskal-Wallis test for three groups. Nominal data were evaluated with the chi-squared test. Correlation analyses were performed with the Spearman coefficient. Additionally, IgA and IgG antibodies against *B. fragilis* and *F. prausnitzii* were analyzed using a linear model with fecal presence of *Bacteriodes* and *F. prausnitzii*, respectively, using diagnosis (patient or control) and the interaction of these two terms as predictors. A two-sided *t*-test was used to test each term for significance, which was set at 0.05. These analyses were performed with SPSS, Version 17 (Chicago, IL, USA) and R (version 3.0.2.)

## Results

### Patients

Clinical and demographic features of the study patients are shown in Table 1. In total 25 children with ERA (11 female, age (median, range) 13, 7 to 19 years) and 13 controls (7 female, age 13, 5 to 17 years) were included in the study. Two of the ERA subjects had IBD. Twenty-one patients, but no controls, were on immunosuppressive therapy.

**Table 1:** **Patient population**

**Feature**	**Enthesitis-related arthritis**	**Controls**
Number	25	13
Age, years, median, range	13.0, 7.0 to 19.0	13.0, 5.8 to 18.0
Male:female, number	11:14	6:7
Body mass index, median, range	20, 16 to 41	23.7, 14.0 to 43.0
Disease duration, years, median, range	2.4, 0 to 8.2	N/A
HLA-B27+, number	9/24 (38%)	0/3
Known inflammatory bowel disease, number	2 (8.0%)	0
Erythrocyte sedimentation rate, median, range	9, 3 to 56	
Medications		
None, number	7 (28%)	13 (100%)
Methotrexate alone, number	2 (8.0%)	
Pentasa alone, number	1 (4.0%)	
Anti-TNF +/− conventional DMARD	15 (60%)	

DMARD, disease-modifying anti-rheumatic drug (methotrexate, leflunomide); TNF, tumor necrosis factor; N/A, not applicable.

### Results of 16S sequencing analysis

**Figure 1:**
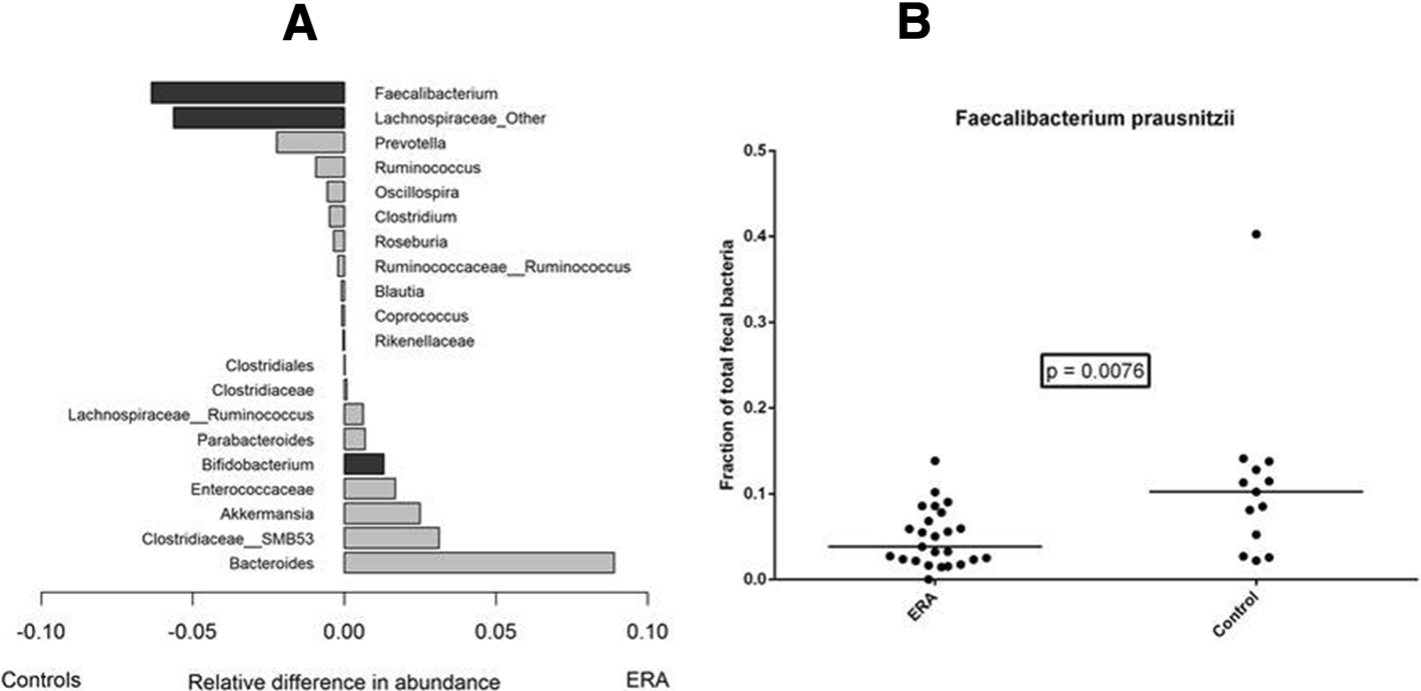
**Decreased abundance of** ***Faecalibacterium*** **genus in enthesitis-related arthritis (ERA) patients compared to controls. (A)** The relative abundance of the top 20 taxa are shown. Although the taxa were evaluated at the genus level, some of the operational taxonomic units (OTUs) were only identified at the family level or above. Negative values (left) indicate higher frequency in controls, and positive values (right) indicate higher frequencies in patients. Statistically significant differences (Mann-Whitney *U*-test) are depicted in a darker gray color. **(B)** The distribution of *F. prausnitzii* levels is shown.

High-quality sequence data were obtained from fecal samples on all 38 children. Following the filtering step, the sequencing depth ranged from 9,000 to 555,000, with a sequence length of 101 base pairs. There were no differences in within-subject (alpha) diversity measurements between the groups (not shown). The taxonomic distribution of the top 20 taxa is shown in Figure 1A. Of these, the only two taxa with statistically significant differences favoring the controls were the *Faecalibacterium* genus (11% versus 5.2%, *P* = 0.005) and unspecified genera of the *Lachnospiraceae* family (12% versus 7.0%, *P* = 0.020). Among those favoring ERA, *Bifidobacterium* showed a modest but statistically significant increase (1.8% versus 0%, *P* = 0.032), and *Bacteroides* demonstrated a larger but statistically non-significant increase (21% versus 11%, *P* = 0.150). Analysis at the species level revealed that 84% of the *Faecalibacterium* sequences represented a single species: *Faecalibacterium prausnitzii*, which constituted 10% of the microbiota of control patients versus 3.8% in the ERA group (*P* = 0.008.) A basic local alignment search tool (BLAST) search of the OTU representing the majority of the unspecified *Lachnospiraceae* family members revealed two matches at 100% identified: *Fusicatenibacter saccharivorans* and *Clostridium clostridioforme*. Thus, it is unclear which of the two organisms drove this apparent difference. Sixty-seven percent of the *Bifidobacterium* sequences represented *B. adolescentis*. The distribution of *F. prausnitzii* levels in patients and controls is depicted in Figure 1B. Additionally, *A. muciniphila* was present in very low quantities in most patients, but was relatively abundant (>2%) in 7/25 (28%) patients, but not in any of the 13 controls (*P* = 0.072, Fisher’s exact). There were no significant associations between the body mass index of the subjects and presence of any of these bacteria (Additional file 1: Table S1.) Among subjects with ERA, HLA-B27 status was not associated with significant differences in the levels of these bacteria (Additional file 1: Table S2).

**Figure 2:**
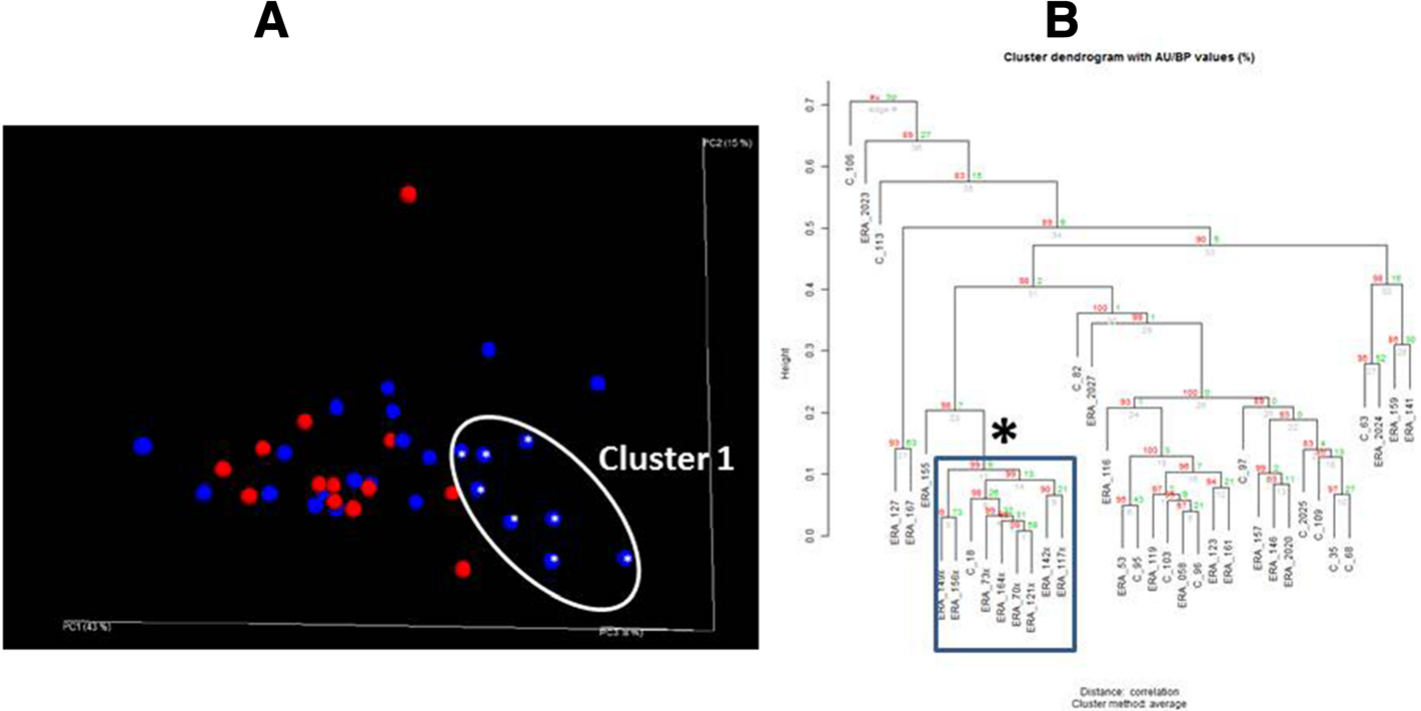
**Spondyloarthritis (SpA) patients have two distinct clusters.** In principal coordinates analysis **(A)** each subject is a separate dot; the closer two subjects are in space, the more similar their sequence data. Enthesitis-related arthritis (ERA) subjects are shown in blue, controls in red. The asterisks over eight of the subjects indicate subjects identified as well with the pvclust bootstrap analysis **(B)**. The same eight subjects are present in the identified cluster.

**Figure 3:**
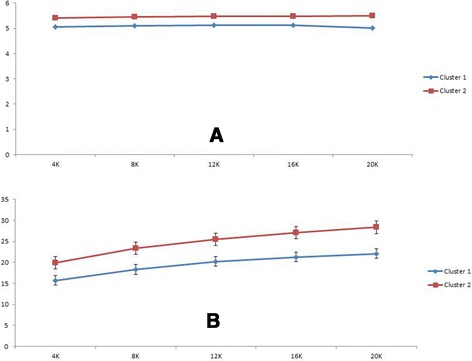
**Alpha diversity metric comparing clusters 1 and 2.** The Shannon index is shown in **A**; this measures species evenness, or the probability of being able to predict the identity of a randomly drawn sequence. The phylogenetic diversity is shown in **B**; this evaluates the length of all branches of the phylogenetic tree of a given population [22].

Thus, at the genus and species level, our data demonstrate some statistically significant differences between patients and controls. Our next step was to assess whether patients and controls may have different overall communities of enteric bacteria. This was evaluated with PCoA (Figure 2A), which demonstrated that while most of the patients clustered with the controls, a set of eight ERA patients formed their own cluster. To confirm the existence of this cluster, we performed bootstrap analysis using pvclust, with the results showing an identical cluster with an AU probability of 99% (Figure 2B.) Comparing the eight ERA patients in cluster 1 with the remainder of the ERA subjects, the former had significantly higher *Bacteroides* levels (32% versus 13%, *P* <0.001), with no difference in the *F. prausnitzii* content (4.7% versus 3.2%, *P* = 0.897). The subgroup of ERA patients with elevated *Bacteroides* content appeared to be under-represented among those patients with high levels (>2%) of *Akkermansia*, which was found in 0/8 of cluster 1 compared to 7/17 (41%) of cluster 2 (*P* = 0.057). Despite the predominance of *Bacteroides* among the subjects in cluster 1, the Shannon index was similar between the two clusters (Figure 3A). However, cluster 1 demonstrated diminished alpha diversity with the phylogenetic diversity metric (3B). The two clusters otherwise appeared similar with respect to most clinical and laboratory features, with the exception that cluster 1 was predominantly female and cluster 2 predominantly male (Table 2.)

**Table 2:** **Comparison of clinical and laboratory features of children in cluster 1 versus cluster 2**

**Feature**	**Cluster 1**	**Cluster 2**	***P*** **-value**
Number	8	17	N/A
Age, years, median, range	13, 7 to 18	13, 7.5 to 19	0.932
Male:female, number	1:7	13:4	0.007
Body mass index, median, range	20, 16 to 33	23, 16 to 41	0.374
Disease duration, years, median, range	1.6, 0 to 6.5	3, 0 to 8.2	0.238
HLA-B27+, number	3/8, 38%	6/16, 38%	1.000
Known inflammatory bowel disease, number	1, 12%	1, 5.9%	1.000
Erythrocyte sedimentation rate, number: median, range	7: 10, 6 to 50	13: 9, 3 to 56	0.536
Medications, number			
None	2, 25%	5, 29%	
Methotrexate alone	0	2, 12%	
Pentasa alone	0	1, 5.9%	
Anti-TNF +/− conventional DMARD	6, 75%	9, 53%	

DMARD, disease-modifying anti-rheumatic drug; TNF, tumor necrosis factor; N/A, not applicable.

**Figure 4:**
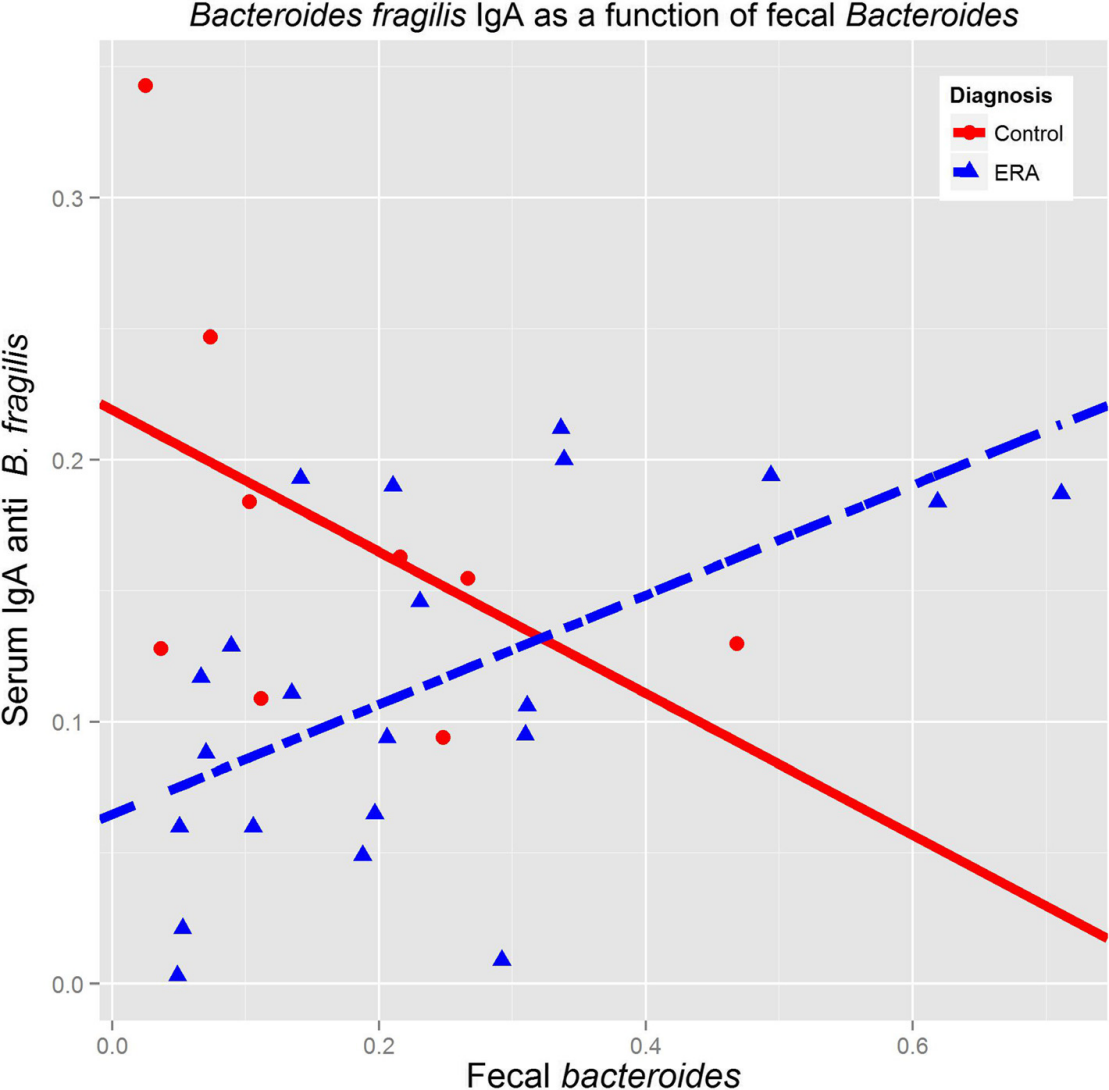
**Divergent relationships between** ***B. fragilis*** **IgA levels and fecal** ***Bacteroides*** **content, between patients and controls.** Enthesitis-related arthritis (ERA) subjects are depicted in blue (broken line); controls are red (solid line).

**Figure 5:**
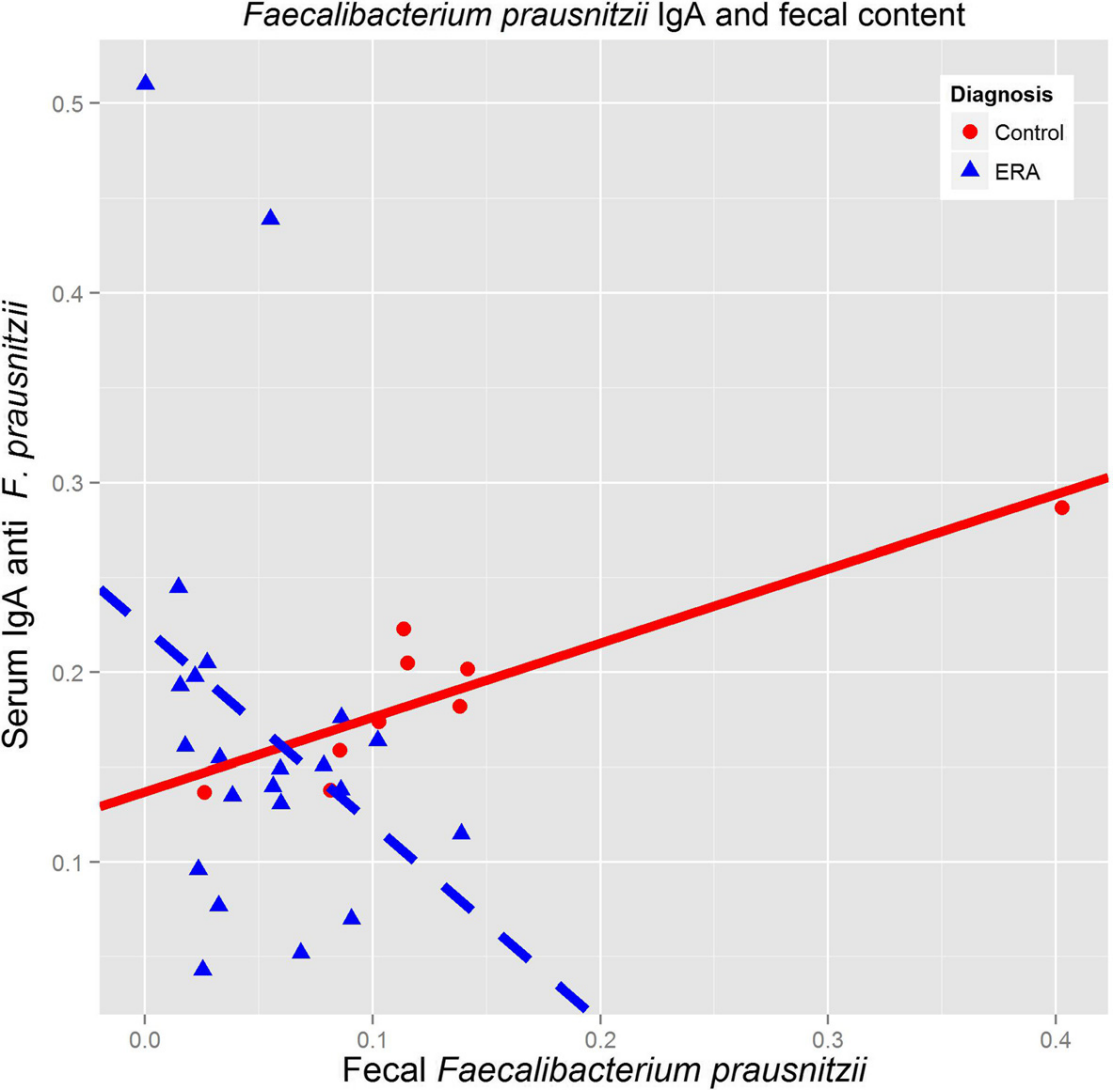
**Divergent relationships between** ***F. prausnitzii*** **IgA levels and fecal** ***F. prausnitzii*** **content, between patients and controls.** Enthesitis-related arthritis (ERA) subjects are depicted in blue (broken line); controls are red (solid line).

Thus, there appear to be significant differences in the populations of enteric bacteria between patients and controls. In order to assess whether the alterations in the frequencies of the bacterial genera could be associated with changes in the immunoreactivity, we performed ELISA against a *Bacteroides* species (*B. fragilis*) and *F. prausnitzii* among the 31 subjects (22 patients and 9 controls) in whom we had serum samples available, including both with IBD. Overall, there were no significant differences in IgA or IgG reactivity against any of the bacteria (Table 3), although HLA-B27+ subjects had elevated antibody production against both (Additional file 1: Table S2.) When broken down by cluster, ERA subjects in cluster 1, characterized by increased *bacteroides*, also demonstrated increased IgA and IgG reactivity against *B. fragilis*, the latter statistically significant at *P* = 0.05 (Additional file 1: Table S3). Interesting trends emerged when we evaluated antibody production against a particular species as a function of the quantity of the associated bacteria. There was a strong correlation (*r* = 0.511, *P* = 0.003) between *Bacteroides* content and IgG antibodies against *B. fragilis* among the group as a whole. In contrast, while the overall correlation between *Bacteroides* content and IgA antibodies was weak (*r* = 0.300, *P* = 0.067), divergent trends emerged within the two groups (Figure 4): ERA patients demonstrated a similar positive correlation (*r* = 0.481, *P* = 0.005), while an inverse although not statistically significant correlation was seen in the control group (*r* = −0.483, *P* = 0.187). Linear modeling of the slopes of the two regression lines demonstrated a statistically significant interaction between IgA levels against *B. fragilis* and diagnosis (*P* = 0.008), not present with IgG (*P* = 0.105). A similar discordance was seen with *F. prausnitzii* (Figure 5), although in this case, patients demonstrated an inverse correlation between IgA antibodies to *F. prausnitzii* and *F. prausnitzii* content (*r* = −0.404, *P* = 0.062), with controls demonstrating a strong positive correlation (*r* = 0.850, *P* = 0.004); as with *B. fragilis*, the slopes of the two curves were statistically significantly different (*P* = 0.039). Neither group demonstrated a strong correlation in either direction between fecal *F. prausnitzii* content and IgG antibodies to the same, although they trended in the same direction (controls: *r* = −0.326, *P* = 0.391; ERA: *r* = −0.113, *P* = 0.615).

**Table 3:** **Median antibody levels (o.d.) against several of the bacteria differentially present between patients and controls**

**Bacteria**	**IgA**			**IgG**		
	**ERA**	**Control**	***P*** **-value**	**ERA**	**Control**	***P*** **-value**
*B. fragilis*	0.11,	0.16,	0.094	0.41,	0.43,	0.536
	0 to 0.21	0.094 to 0.343		0.095 to 1.3	0.20 to 1.6	
*F. prausnitzii*	0.15,	0.18,	0.112	0.45,	0.46,	0.742
	0.043 to 0.51	0.14 to 0.29		0.21 to 1.6	0.22 to 1.6	

ERA, enthesitis-related arthritis. Values are median (min - max). o.d. = optical density.

## Discussion

The human intestine is colonized with an estimated 100 trillion bacteria, a process that begins shortly after birth [25]. It is becoming increasingly clear that these bacteria play important roles in immune function as well as in a variety of autoimmune and inflammatory disorders [25]. One condition with important microbial contributions is IBD, as evidenced by aberrant microbial content [26], therapeutic response to antibiotics in CD [27], and abnormal innate and adaptive immunologic responses to enteric bacteria [28]. There is also interest in the role of the microbiota in SpA [29], with a previous study showing possible decreases in the *Bacteroides-Provotella* and *Clostridium leptum* groups, with an increase in *Bifidobacterium* among AS patients compared to controls, although the overall differences were not statistically significant [8].

In this study, four candidate organisms are identified as being potentially associated with ERA. Decreased *F. prausnitzii*, was seen in the patients compared to controls. This finding has previously and consistently been reported in patients with IBD [30-32], and may be of particular significance in light of its reported anti-inflammatory effects [33]. It may also be consistent with the previous finding of decreases in the *Clostridium leptum* group in AS patients [8], as both *C. leptum* and *F. prausnitzii* are in the *clostridiaceae* family. *F. prausnitzii* is an important producer of butyrate [34], which may have anti-inflammatory properties [35]; there was no compensatory increase in other butyrate-producing bacteria, such as the *Eubacterium* and *Roseburia* genera (data not shown) [34,36]. These findings underscore the pathogenic similarities between IBD and ERA, as *F. prausnitzii* was not reduced in a study of newly diagnosed RA patients [37]. Additional evidence of microbial similarities between IBD and ERA comes from the study by Frank *et al*. [32], which showed not only decreased *F. prausnitzii* in the IBD patients, but also decreases in the *Lachnospiraceae* family. In particular, there may have been a decrease in *Clostridium clostridioforme,* which was one of the two potential organisms associated with the dominant OTU in the *Lachnospiraceae* family significantly under-represented in the ERA patients. That these differences may result from genetic differences between the populations, rather than from the disease process itself, was suggested by a study showing that *NOD2* and *ATG16L1* genotypes were associated with altered microbial populations even with adjustment for diagnosis of IBD [38]. This information was not available for our patients.

A novel finding of this study is that both *Bacteroides* species and *Akkermansia muciniphila* were found to be associated with disease states in largely non-overlapping subsets of ERA patients. These subsets were similar with respect to most clinical and laboratory features, aside from cluster 1 being predominantly female, and cluster 2 predominantly male. The potential relevance of this distinction awaits further exploration. *B. fragilis* is considered to have regulatory effects through its polysaccharide A [39], although the enterotoxigenic strain of *B. fragilis* has been linked to IBD [40]. Additionally, in both a rat and a mouse model of SpA abrogated by the germ-free state, addition of a consortium of bacteria that includes *bacteroides* species results in active disease, with *B. vulgatus* specifically identified as a causative agent in the rat model [41,42]. Furthermore, as indicated above, abnormal humoral and T cell responses to *bacteroides* has been reported in AS patients [8,12]. The data reported herein indicated that up to one third of ERA patients have markedly elevated *Bacteroides* content, while a second subset had similar overall patterns of enteric bacteria to the controls, with the exception of lower *Faecalibacterium*. However, 7/17 of the ERA group with normal *Bacteroides* content, compared to 0/8 of the group with elevated *Bacteroides* content, carried increased frequencies of *A. muciniphila*. First described in 2004, it was originally isolated based upon its capacity to thrive on intestinal mucin, suggesting that high quantities could potentially lead to a defect in the intestinal wall barrier function [43]. Although a European study indicated that *A. muciniphila* typically comprises 1 to 3% of the intestinal microbiota [44], it is evidently less common in the United States, or at least in our patient population, and there may be regional differences in the overall microbiota that could result in patients in this region being more susceptible to a pro-inflammatory effect of this bacterium. This is the first study suggesting a potential role in inflammatory disease in humans, although a recent study of mice infected with *Salmonella* demonstrated increased histologic inflammatory scores and mRNA levels of inflammatory markers in mice that also received *A. muciniphila* [45].

Finally, elevated *Bifidobacterium*, mostly *B. adolescentis*, was also identified among ERA patients. There are mixed data with respect to this genus in the context of IBD, with prior studies both showing elevated [31] as well as decreased presence [46]. Elevated *bifidobacterium* was also seen in the study of AS patients [8]. Thus, overall, ERA patients displayed a microbiota profile similar to that published for IBD patients with respect to lower levels of *F. prausnitzii* and possibly of *Clostridium clostridioforme,* as well as alterations in *B. adolescentis.* Our findings are also consistent with those reported in AS patients, despite substantial differences in age and disease duration in the current study compared to the previous [8].

IgA functions in part to protect the host against commensal organisms [47,48]. It also has a regulatory role, with downstream responses to IgA binding dependent upon a variety of factors, including signaling events through the IgA receptor and the location of the dendritic antigen presenting cells [49,50]. Thus, the interpretation of discordant correlations between IgA and 16S levels between patients and controls is clouded by the multiple functions of IgA. In the case of *B. fragilis*, the relationship between the IgA and the sequence data may represent invasion of the gut wall by the bacterium, triggering systemic immune responses. Alternatively, in light of its potential regulatory role, the inverse relationship between IgA against *F. prausnitzii* and the sequence data may represent a failure of the system to generate a regulatory T cell response normally thought to be induced by this species [33]. Studies of T cell function are required to evaluate these possibilities.

This study has several limitations. The patient numbers are limited, and they included subjects with newly diagnosed as well as treated arthritis. However, a study of IBD patients showed that while antibiotics and aminosalicylates had important effects on the microbiota, immunosuppressive therapies appeared to have less of an impact [51]. Exclusion of patients with IBD did not impact the trends shown herein, although the association between *F. prausntzii* levels and antibodies among ERA patients was less robust (Additional file 1: Tables S4, S5).

An additional limitation is that information on the diet of these subjects was unavailable on most of the patients. However, body mass index data were not predictive of the levels of any of the bacteria identified in this study (Additional file 1: Table S1). Furthermore, although the controls did have higher BMI compared to the patients (Table 1), at the phylum level, the *Firmicutes*: *Bacteroidetes* ratio was similar (2.97, 0.26 to 30 in ERA patients compared to 3.46, 1.0 to 26 in controls, *P* = 0.199, and there was a small albeit statistically insignificant inverse relationship between body mass index and the *Firmicutes*/*Bacteroidetes* ratio among the group as a whole (*r* = −0.140, *P* = 0.401), the opposite direction that would be expected if weight were the driving factor [52]. Finally, there was no external validation of the differences in bacterial content observed in this study, nor was there objective measurement of intestinal inflammation (for example, fecal calprotectin.) This is, nevertheless, the first comprehensive evaluation of the microbiota in pediatric or adult SpA, confirming a potential role for insufficient protective *F. prausnitzii* in the pathogenesis of ERA and introducing potential novel bacteria as associative agents in ERA. These findings suggest altering the gut microbiota may be beneficial in children with ERA. Future directions will extend these findings in larger cohorts and also identify the nature of the adaptive (antibody and T cell reactivity) immunologic response against these enteric commensal organisms.

## Conclusions

Our studies provide further evidence of a pathogenetic link between IBD and SpA by showing a similar decrease in fecal *F. prausnitzii* content in ERA patients as had previously been reported in subjects with IBD. In addition, we introduce two organisms, *Bacteroides* genus and *A. muciniphila*, as potential associative agents in subsets of children with ERA. Finally, our data suggest that alterations to IgA reactivities against commensal organisms may play a role in ERA.

## Additional file

Additional file 1: Table S1. Correlation between body mass index and sequencing data. **Table S2.** Effect of HLA-B27 status on sequencing data and antibody titers, among subjects with enthesitis-related arthritis (ERA). **Table S3.** Antibody titers by ERA cluster. **Table S4.** Sequencing data and antibodies titers, following exclusion of patients with inflammatory bowel disease (IBD). **Table S5.** Correlation between sequencing data and antibody titers, following exclusion of subjects with IBD.

## Abbreviations

AS: ankylosing spondylitis
AU: approximate unbiased
BMI: body mass index
BP: bootstrap probability
CD: Crohn’s disease
CoA: Children’s of Alabama
DMARD: disease-modifying anti-rheumatic drug
ELISA: enzyme-linked immunosorbent assay
ERA: enthesitis-related arthritis
IBD: inflammatory bowel disease
OTU: operational taxonomic unit
PBS: phosphate-buffered saline
PCoA: principal coordinates analysis
QIIME: Quantitative Insight Into Microbial Ecology
RA: rheumatoid arthritis
rDNA: ribosomal DNA
SpA: spondyloarthritis
TNF: tumor necrosis factor
