# Supplementary material for: Altered microbiota associated with abnormal humoral immune responses to commensal organisms in enthesitis-related arthritis
Source: Arthritis Res Ther. 2014 Nov 30;16(6):486. doi: 10.1186/s13075-014-0486-0 (PMC4272554; doi:10.1186/s13075-014-0486-0)
Supplement: Additional file 1: Table S1. — Correlation between body mass index and sequencing data. Table S2. Effect of HLA-B27 status on sequencing data and antibody titers, among subjects with enthesitis-related arthritis (ERA). Table S3. Antibody titers by ERA cluster. Table S4. Sequencing data and antibodies titers, following exclusion of patients with inflammatory bowel disease (IBD). Table S5. Correlation between sequencing data and antibody titers, following exclusion of subjects with IBD. [file 13075_2014_486_MOESM1_ESM.docx]

Table S1. Correlation between body mass index and sequencing data

|  | ERA | Controls |
| --- | --- | --- |
| *Bacteroides* | r = 0.117, p = 0.578 | r = 0.154, p = 0.616 |
| *F. prausnitzii* | r = -0.360, p = 0.077 | r = 0.423, p = 0.150 |
| *Akkermansia* | r = -0.150, p = 0.475 | r = -0.256, p = 0.399 |

Table S2. Effect of HLA-B27 status on sequencing data and antibody titers, among subjects with ERA

| Variable | HLA-B27+ | HLA-B27- | p |
| --- | --- | --- | --- |
| n | 9 | 15 | N/A |
| **Sequencing data** |  |  |  |
| *Bacteroides* | 0.29, 0.07 – 0.49 | 0.21, 0.031 – 0.71 | 0.379 |
| *F. prausnitzii* | 0.050, 0 – 0.09 | 0.033, 0.015 – 0.14 | 0.815 |
| *Akkermansia* > 2% | 1/9, 11% | 6/15, 40% | 0.191 |
| **IgA reactivity** |  |  |  |
| *B. fragilis* | 0.06, 0 – 0.21 | 0.12, 0 – 0.20 | 0.446 |
| *F. prausnitzii* | 0.16, 0.05 – 0.51 | 0.15, 0.043 – 0.44 | 0.972 |
| **IgG reactivity** |  |  |  |
| *B. fragilis* | 0.32, 0.19 – 0.63 | 0.57, 0.095 – 1.3 | 0.037 |
| *F. prausnitzii* | 0.40, 0.21 – 0.51 | 0.59, 0.33 – 1.60 | 0.025 |

Table S3. Antibodies titers by ERA Cluster

| Variable | Cluster 1 | Cluster 2 | p |
| --- | --- | --- | --- |
| n | 8 | 17 | N/A |
| **IgA reactivity** |  |  |  |
| *B. fragilis* | 0.19, 0.009 – 0.20 | 0.09, 0.003 – 0.21 | 0.110 |
| *F. prausnitzii* | 0.15, 0.070 – 0.51 | 0.15, 0.043 – 0.44 | 0.764 |
| **IgG reactivity** |  |  |  |
| *B. fragilis* | 0.54, 0.37 – 1.3 | 0.33, 0.095 – 0.74 | 0.050 |
| *F. prausnitzii* | 0.58, 0.21 – 0.84 | 0.42, 0.26 – 1.6 | 0.165 |

Table S4. Sequencing data and antibodies titers, following exclusion of subjects with IBD

| Variable | ERA | Controls | p |
| --- | --- | --- | --- |
| n | 25 | 13 | N/A |
| **Sequencing data** |  |  |  |
| *Bacteroides* | 0.21, 0.003 – 0.71 | 0.11, 0.025 – 0.47 | 0.199 |
| *F. prausnitzii* | 0.05, 0 – 0.14 | 0.10, 0.022 – 0.40 | 0.018 |
| *Akkermansia* > 2% | 7/23, 30% | 0/13 | 0.034 |
| **IgA reactivity** |  |  |  |
| *B. fragilis* | 0.11, 0.003 – 0.21 | 0.16, 0.094 – 0.34 | 0.086 |
| *F. prausnitzii* | 0.14, 0.043 – 0.51 | 0.18, 0.14 – 0.29 | 0.063 |
| **IgG reactivity** |  |  |  |
| *B. fragilis* | 0.41, 0.095 – 1.3 | 0.43, 0.20 – 1.6 | 0.533 |
| *F. prausnitzii* | 0.45, 0.21 – 1.6 | 0.46, 0.22 – 1.6 | 0.790 |

Table S5. Correlation between sequencing data and antibody titers, following exclusion of subjects with IBD

|  | ERA | Controls |
| --- | --- | --- |
| *Bacteroides* / IgA | r = 0.627, p = 0.003 | r = -0.483, p = 0.187 |
| *Bacteroides* / IgG | r = 0.466, p = 0.039 | r = 0.533, p = 0.139 |
| *F. prausnitzii* / IgA | r = -0.302, p = 0.195 | r = 0.850, p = 0.004 |
| *F. prausnitzii* / IgG | r = -0.071, p = 0.767 | r = -0.326, p = 0.391 |
